# Transcriptome Analysis Reveals Key miRNA–mRNA Pathways in Ovarian Tissues of Yunshang Black Goats With Different Kidding Numbers

**DOI:** 10.3389/fendo.2022.883663

**Published:** 2022-05-19

**Authors:** Yufang Liu, Zuyang Zhou, Siwu Guo, Kunyu Li, Peng Wang, Yekai Fan, Xiaoyun He, Yanting Jiang, Rong Lan, Shuangzhao Chen, Shenghong Dai, Qionghua Hong, Mingxing Chu

**Affiliations:** ^1^ Key Laboratory of Animal Genetics, Breeding and Reproduction of Ministry of Agriculture and Rural Affairs, Institute of Animal Science, Chinese Academy of Agricultural Sciences, Beijing, China; ^2^ College of Life Sciences and Food Engineering, Hebei University of Engineering, Handan, China; ^3^ Yunnan Animal Science and Veterinary Institute, Kunming, China; ^4^ Wuhan Frasergen Bioinformatics Co., Ltd., Wuhan, China

**Keywords:** goat, reproduction trait, steroid hormone secretion, *JAK3*, chi-miR-493-3p

## Abstract

The granulosa cell (GC) is the basic functional unit of follicles, and it is important for promoting follicle growth and sex hormones, as well as growth factor secretion in the process of reproduction. A variety of factors influence granulocyte proliferation, yet there are still many gaps to be filled in target and non-coding RNA regulation. In our study, the differentially expressed (DE) mRNAs and miRNAs were detected by using RNA-seq, and we constructed a mRNA–miRNA network related to goat prolificacy. Then, the goat primary GCs were isolated from the follicle for the function validation of candidate genes and their regulator miRNAs. A total of 2,968 DE mRNAs and 99 DE miRNAs were identified in the high- and low-prolificacy goat by RNA-seq, of which there were 1,553 upregulated and 1,415 downregulated mRNAs, and 80 upregulated and 19 downregulated miRNAs, respectively. *JAK3* was identified as highly expressed in the low-prolificacy goats (3 times higher than high-prolificacy goats), and the integrated analysis showed that chi-miR-493-3p was a potential regulator of *JAK3*. The analysis of Kyoto Encyclopedia of Genes and Genomes (KEGG) showed that JAK3 was involved in the PI3K-Akt signaling pathway, the Jak-STAT signaling pathway, and signaling pathways regulating pluripotency of stem cells. In particular, the PI3K-Akt signaling pathway was a typical pathway for cell proliferation, differentiation, apoptosis, and migration. We found that the chi-miR-493-3p targets *JAK3* directly *via* RT-qPCR, dual fluorescence assays, and Western blot. Furthermore, the expression of JAK3 was significantly decreased by the chi-miR-493-3p mimic and increased by the chi-miR-493-3p inhibitor. The CCK-8 assay showed that overexpression of *JAK3* promoted cell proliferation, while inhibiting *JAK3* had the opposite effect. The expression of cell proliferation markers CDK4 and cyclin D2 also showed the same results. Moreover, the enzyme-linked immunosorbent assay showed that steroid hormones E_2_ and PROG were increased by overexpressing JAK3 and decreased by inhibiting JAK3. Therefore, our results identified a chi-miR-439-3p-JAK3 regulatory pathway, which provided a new insight into the GC proliferation and prolificacy of goat.

## Introduction

In female livestock, the fertility trait is a major index because of its economic value. The major rate-limiting factor to fertility is the decline of oocyte quality, and the oogenesis and folliculogenesis proceed in parallel within the ovary (1, 2). As an important endocrine gland and gonad, the ovary communicates with other tissues by secreting steroid hormones, cytokines and microRNAs, and signaling pathways so as to optimize the follicular environment for oocyte maturation (3). In this process, granulosa cells (GCs) are crucial in the development of follicle and oocyte, ovulation and luteinization, and steroid hormone secretion including progesterone and estrogen, which provide an important microenvironment for follicle growth (4). Hence, GC proliferation is essential for female livestock, and the molecular mechanism underlying the process of GC proliferation needs to be revealed.

The nutritional hormones follicle-stimulating hormone (FSH), luteinizing hormone (LH), and angiotensin II (Ang II)/potassium (K^+^) control the overall rate of steroid hormone production in the ovary with acute and chronic regulation (5, 6). In female animals, the process of progesterone and estrogen synthesis was controlled by FSH in ovarian GCs, while LH regulates the synthesis of progesterone in luteinized GCs and luteal cells, and the production of androgens in ovarian interstitial cells of the corpus luteum (7). Many genes participated in the steroid hormone production in the ovary; for example, StAR, P450arom (CYP19A1), P450scc (CYP11A1), and 3βHSD2 are all expressed in two cell types (theca and GCs) that are able to produce pregnenolone/progesterone from cholesterol substrate (8). In recent years, post-transcription regulation had become an important means to influence the expression of numerous economic traits. MiRNAs, a range of conservative non-coding RNA molecules that are 18–25 nucleotides (nts) in length, participated in a series of ovarian physiological events including ovarian growth and development, proliferation of GCs, and steroid hormone regulation (9–12). It is well known that miRNAs are mainly regulated through the binding of mature miRNA/RISC (induced silencing complex) complexes to complementary sites in the target mRNA, which negatively regulates gene expression. In the expression profiling study of mouse preantral GCs, miRNA-224 performs a regulatory function in TGF-β1-induced GC proliferation and release of estradiol by targeting Smad4 (10). The miRNA-378 has also been demonstrated to negatively regulate the production of estradiol and oocyte maturation in pig GCs *via* targeting estradiol synthase using loss-of-function and gain-of-function strategies (11, 13). In goat, miR-101-3p, miR-200a, miR-141, and miR-206 have been reported to affect the GCs’ development (14–17); however, the function of many miRNAs related to GC development and ovarian growth remains unknown.

In this study, mRNA and miRNA sequencing were used to validate the differentially expressed (DE) mRNAs and miRNAs in the ovarian tissues of Yunshang black goat, a native high-prolificacy goat breed in China. The miRNA–mRNA network was constructed to screen the potential molecular pathways regulating high-prolificacy traits in goat. We selected JAK3 from the downregulated DEGs, a potential target of chi-miR-493-3p, to further investigate its function involvement in ovarian development. Then, we assessed the regulatory effects and molecular mechanism of chi-miR-493-3p-JAK3 on goat GC proliferation and steroid hormone synthesis *in vitro*. The current study provided basic data for understanding the impact of chi-miR-493-3p and JAK3 on goat ovarian development.

## Materials and Methods

### Ethics Statement

All experimental procedures mentioned in this study were approved by the Science Research Department (responsible for animal welfare issues) of the Institute of Animal Sciences, Chinese Academy of Agricultural Sciences (IAS-CAAS) (Beijing, China). Ethical approval was provided by the animal ethics committee of IASCAAS (No. IAS2021-25).

### Sample Preparation and RNA Extraction

In this study, ten female native domestic goats in a goat farm in Yunnan Province, named Yunshang black goat, were used. The height, weight, and age (2–3 years old) of goats, with no significant differences, were randomly selected and grouped into a high-prolificacy group (*n* = 5, mean kidding number = 3.4 ± 0.42, H group) and a low-prolificacy group (*n* = 5, mean kidding number = 1.8 ± 0.27, L group) according to their kidding number records. All animals were treated for estrus synchronization. This was done by treating female goats with progesterone suppositories for 16 days and collecting ovarian tissues at slaughter 11 days after stopping the hormones. The ovarian tissues were collected from goats and then immediately frozen in liquid nitrogen and stored at −80°C until RNA was extracted.

According to the manufacturer’s instruction, the total RNA of 10 ovarian samples was isolated by TRIzol reagent (Invitrogen, Carlsbad, CA, United States) for RNA sequencing (RNA-seq). The NanoDrop 2000 spectrophotometer (Thermo Scientific, Wilmington, DE, United States) was used to assess the purity and concentration of the RNA samples, and the degree of degradation and contamination was monitored by standard denaturing agarose gel electrophoresis. An RNA Nano 6000 Assay Kit of the Agilent Bioanalyzer 2100 system (Agilent Technologies, Palo Alto, CA, United States) was used to assess the integrity of RNA.

### Library Preparation and Sequencing

Ten cDNA libraries of the ovarian tissues from two Yunshang black goat groups with high- and low-prolificacy kidding numbers (H and L group) were constructed. A total of 3 µg of RNA from each sample was used as input material for the miRNA library and cDNA library. First, an Epicentre Ribo-Zero™ rRNA Removal Kit (Epicentre, Madison, WI, United States) was used to remove the ribosomal RNA, and the ethanol precipitation was used to clean up the rRNA-free residues. Secondly, following the manufacturer’s recommendations, the NEBNext^®^ Ultra™ Directional RNA Library Prep Kit for Illumina^®^ (NEB, Ipswich, MA, United States) was used in the rRNA-depleted RNA to generate sequencing libraries. Finally, an AMPure XP system was used to purify the products, and an Agilent Bioanalyzer 2100 system was used to assess the quality of the libraries. An Illumina Hiseq 2500 platform was used to sequence the libraries, which generated 150 bp of paired-end reads.

The fastq format raw data were initially processed by in-house scripts. Subsequently, the raw reads of Illumina sequencing were used for the removal of reads containing adapter, ploy-N, and low-quality reads, and the quality value *Q* ≤ 20 was maintained over 50%. The size of clean data was calculated depending on the Q20, Q30, and GC content. Because all downstream analyses were based on clean data, they must be maintained at a high level of quality. We directly downloaded the goat reference genome and the files of gene model annotation from the genome website. Bowtie v2.2.3 was used to build the index of the reference genome, and TopHat v2.0.12 was used to align the pair of clean reads to the reference genome. The known and novel transcripts from the TopHat alignment results were constructed and identified by the Cufflinks v2.1.1 Reference Annotation Based Transcript (RABT) assembly method. According to the length of the gene and read counts mapped to the gene, the fragment per kilobase per million reads (FPKM) for each gene was calculated (18).

In the subsequent analyses, the clean high-quality reads with lengths of 18–35 nt were used. The small RNA tags were mapped to Bowtie21 reference sequences, which were used to find out the known miRNAs. Using miRBase22.0 as a reference database, the potential miRNAs were obtained by the modified software mirdeep2 and srna-tools-cli, and then their secondary structures were mapped (19). In order to remove tags derived from repetitive sequences, protein-coding genes, tRNAs, rRNAs, snoRNAs, and snRNAs, the tags of small RNA were mapped to the Repeat Masker or Rfam databases or to data from the specified species. The characteristics of the miRNA precursor hairpin structures could be used to predict the novel miRNAs. The secondary structure Dicer cleavage sites and the minimum free energy of unannotated small RNA tags in the previous steps of novel miRNAs were predicted by the software miREvo and mirdeep2 (19, 20).

### Identification of Differentially Expressed mRNA and miRNA

The mRNA expression levels in the constructed libraries of ovarian tissues were estimated according to the sequencing data of Illumina, based on the values of FPKM. The fold change (FC) for mRNA between the H and L groups was calculated according to comparisons of the combination. The DESeq R package (1.8.3) was used to analyze the DE mRNAs from the two groups. The DE genes were adjusted by *q* < 0.05 and |log_2_FoldChange| > 1 of DESeq. According to the values of normalized transcripts per kilobase per million reads (TPM), the differentially expressed miRNAs (DEMs) were analyzed by the DEGseq R package. The *q*-values were adjusted by *p*-values, and the significant DEMs were set by default by the threshold *q*-values < 0.01 and |log_2_FoldChange| > 1.

### Integrated Analysis of Differentially Expressed miRNA and mRNA

The miRNA and transcriptome profiles of the two groups with different kidding numbers were constructed by using the ovarian tissues from Yunshang black goats. To analyze the interactions between miRNAs and mRNAs, first, miRanda (21) was used to predict miRNA target genes with psRobot_tar in psRobot (22). Then, according to miRNA expression profiles and transcriptome data, the interactions of miRNA–mRNA were calculated, and Pearson correlation analysis was used to determine the negative correlation of miRNA–mRNA pairs. Finally, the “GeneSymbol” as a unique identifier was created for all genes/transcripts involved in all these analyses.

### GO and KEGG Pathways of Targeting Gene Analysis

The Gene Ontology (GO2) with the software GOSeq (Release2.12) (23) and Kyoto Encyclopedia of Genes and Genomes (KEGG) pathway analysis with the software KOBAS (v2.0) (24) were used to annotate and classify mRNAs and enriched genes among miRNA-targeted mRNAs to visualize data. The enrichment on GO terms or KEGG pathways with corrected *p*-values (*t*-test) < 0.05 was indicated as significant.

### Protein–Protein Interaction Network Analysis of DEGs

Using the STRING database (https://string-db.org/), the PPI analysis of DE genes was known and the protein–protein interactions were predicted. The STRING database4 (organism: *Capra hicus*) was used for PPI analysis of DEGs. The networks were constructed by the extracted target gene list from the database. In addition, the sequences of target genes and the selected reference protein were aligned by Blastx (v2.2.28), and then the known interaction of selected reference species was used to build the networks.

### Validation of Differentially Expressed mRNA and miRNA by Real-Time Qualitative PCR

For the expression analysis of genes, a PrimeScript™ RT reagent kit (TaKaRa) and a miRcute Plus miRNA First-Strand cDNA Kit (TIANGEN, Beijing, China) were used for reverse transcription of mRNA and miRNA according to the manufacturer’s protocol, respectively. RT-qPCR was performed using a RocheLight Cycler^®^480 II system (Roche Applied Science, Mannheim, Germany) with SYBR Green qPCR Mix (TaKaRa, Dalian, China) for mRNAs and the miRcute Plus miRNA qPCR Kit (TIANGEN, Beijing, China) for miRNAs. The RT-qPCR for mRNAs was conducted with the following procedure: initial denaturation at 95°C for 5 min, followed by 40 cycles of denaturation at 95°C for 5 s, then annealing at 60°C for 30 s. The RT-qPCR for miRNA was conducted as follows: initial denaturation at 95°C for 15 min, followed by 40 cycles of denaturation at 94°C for 20 s, then annealing at 60°C for 34 s. The 2*^−ΔΔ^* ^Ct^ method was used to analyze the data. The goat *PRL19* and U6 were used as reference genes for normalization of the target gene and miRNA data. The primer sequences used for RT-qPCR are listed in **Supplementary Table S1**.

### Phylogenetic Tree Construction

The sequencing results were subjected to a BLAST search (NCBI, http://blast.ncbi.nlm.nih.gov/Blast.cgi) to retrieve homologous gene sequences of *JAK3*. Multiple nucleic acid sequences were compared using DNAMAN software, and a phylogenetic tree was constructed using the Neighbor-Joining method in MEGA7 software.

### Western Blotting Assay

The total protein of ovary tissues was isolated by the proteinase inhibitor-containing lysis buffer. The 4% SurePAGE gel (GenScript, Nanjing, China) was used to separate the equivalent amounts of protein. The separated proteins were placed onto a PVDF membrane (Pall, Mexico) after electrophoresis, and then blocked with sealing solution (Tiangen, Beijing, China). The blocked membrane was incubated overnight at 4°C with anti-JAK3 (1:500; Santa Cruz, Cambridge, UK), anti-CDK4 (1:1,000), anti-cyclin D2 (1:1,000) (all from Cell Signaling Technology, US), and GAPDH (1:2,000; Proteintech, Chicago, IL, USA). After rinsing 3 times with Tris-buffered saline/Tween, the corresponding HRP-labeled Goat Anti-Rabbit IgG (1:1,000; Beyotime) or HRP-labeled Goat Anti-Mouse IgG (1:1,000; Beyotime) was used to incubate the membranes for 1 h at room temperature. The Enhanced Chemiluminescent Reagent (Beyotime) was used to visualize the protein blots.

### Cell Culture, Vector Construction, and Transfection

Following previously described methods, primary goat GCs were isolated from the follicles of ovarian tissues in the follicular phase (25). The diameter of the GCs in the follicular phase was over 3.5 mm. The isolated cells were seeded in 6-mm plates and maintained in a complete medium [DMEM/F12 (1:1), 10% FBS, and 1% penicillin/streptomycin] as described by Yang et al. (26). When the cell confluence was >90%, the cells were transferred to 10-mm plates for the next experiment.

The region of *JAK3* 3’ UTR containing the predicted target site, including wild type (WT) and mutant type (MUT), was cloned into a pmiR-RB-Report vector [isolated using *Xho*I and *Not*I, respectively (Takara, Dalian, China)]. The recombinant vectors were named pmiR-RB-JAK3-WT/pmiR-RB-JAK3-MUT. The inserted wild/mutant sequence, miRNA mimic, and mimic-NC were synthesized by the RiboBio company (Guangzhou, China). The coding region of JAK3 was cloned into a pcDNA3.1 expression vector, and the recombinant vectors were named pcDNA3.1-JAK3 and pcDNA3.1-NC. siRNA-JAK3, siRNA-NC, and the chi-miR-493-3p mimic and inhibitor were synthesized by Genewiz (Suzhou, China). A human renal epithelial cell line, HEK293T, was used to validate the target of miRNA. Cells were seeded into 24-well plates. Co-transfection with 200 ng of target JAK3-3’UTR-WT or target JAK3-3’UTR-MUT and 10 µl of miRNAs mimic or mimic-NC was performed with Lipofectamine 2000 (Invitrogen, USA). Goat primary GCs were seeded into 6-well plates and transfected with pcDNA3.1-JAK3, siRNA-JAK3, and the chi-miR-493-3p mimic or inhibitor (RiboBio, Guangzhou, China); meanwhile, their NC was used as negative control. Furthermore, after transfection, cells were collected for transfection efficiency analysis, and the proliferation of cells and the expression of genes were examined after 48-h transfection.

### CCK-8 Assay

The goat GCs with an added growth medium were seeded in 96-well cell culture dishes. When the cell density reached the appropriate level, the cells were transfected. The proliferation of cells after 0, 6, 12, 24, and 48 h of transfection was detected according to the instruction protocol of CCK-8 Kit (Vazyme, Nanjing, China), and the multi-mode micropore detection system (EnSpire, Perkin Elmer, USA) was used to detect the absorbance at 450 nm.

### Dual-Luciferase Reporter Assay

HEK293T cells and GCs were detected by the dual luciferase assay. HEK293T cells and GCs were evenly seeded in a 24-well cell culture dish. When the cells reached the appropriate density, they were co-transfected with pmiR-RB-JAK3-WT (wild type) or pmiR-RB-JAK3-MUT (mutant type) and chi-miR-493-3p mimic or mimic-NC. After 48-h transfection, according to the instructions of the double luciferase detection kit (Vazyme, Nanjing, China), the luciferase activity was detected by the multi-mode micropore detection system (EnSpire, Perkin Elmer, USA).

### The Hormone Level Detected by Enzyme-Linked Immunosorbent Assay

ELISA was used to detect the levels of E_2_ and PROG in GCs with different treatment according to the protocol of the E_2_ ELISA Kit and the PROG ELISA Kit (Enzyme-linked Biotechnology Co., Ltd., Shanghai, China). The goat GC culture supernatant was transfected, and pcDNA3.1-JAK3, siRNA-JAK3, and their NC were centrifuged at 2000 × *g* for 5 min at room temperature. Then, 10 μl of the sample supernatant was collected and added to the bottom of the plate wells along with 40 μl of sample diluents and 100 μl of enzyme-labeling reagents. After sealing the plates with sealing film, they were incubated at 37°C for 60 min. Finally, we followed standard procedures of washing, color development, and termination of the plates. The absorbance at 450 nm was determined by using a Fluorescence/Multi-Detection Microplate Reader (Bio Rad, USA).

### Statistical Data Analysis

The GraphPad Prism (version 5.0) software (San, Diego, CA, United States) was used to analyze the results of RT-qPCR and for graphing. The paired *t*-tests were used to test the statistical significance of the data. The results were expressed as the means ± SEM of three replicates, and the statistical significance was expressed as *p*-values < 0.05 (*p < 0.05; **p < 0.01).

## Results

### Summary of mRNA and miRNA Library Sequencing of Goat Ovaries

**Figure 1:**
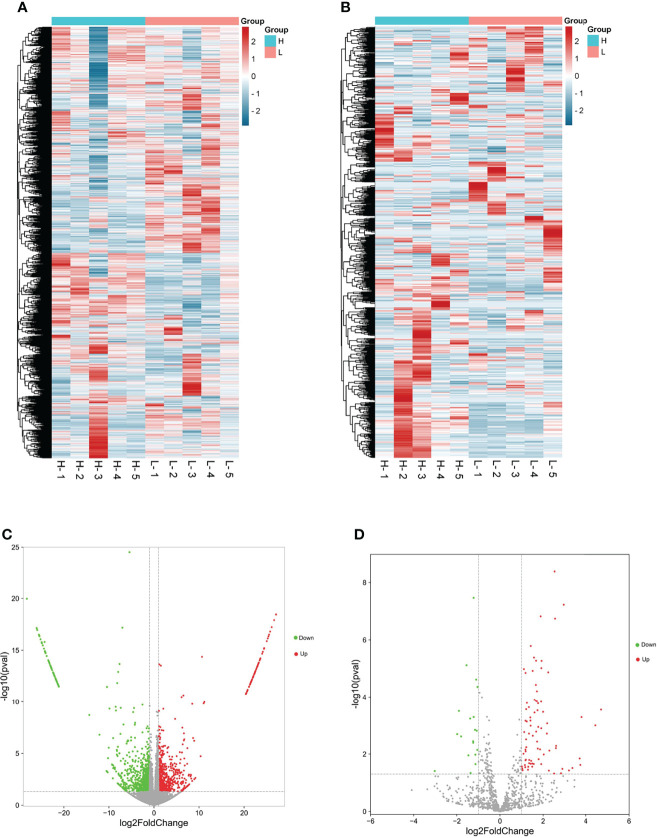
Overall miRNA and mRNA prolife in goat ovary by RNA-seq. **(A, C)** were the differentially expressed mRNA cluster and Volcano diagram, respectively; **(B, D)** were the differentially expressed miRNA cluster and Volcano diagram, respectively.

The cDNA and small RNA libraries were contrasted by RNA-seq with 10 goat ovaries, and after quality screening, 115 million and 26 million clean read pairs were obtained from all cDNA and small RNA libraries, respectively, of which 96.99% of mRNA and 94.88% of miRNA reads from the samples, on average, were mapped to the goat genome (*Capra hircus*, ARS1) (**Table 1**). A series of filter criteria were used to determine mRNA and miRNA candidates. A total of 28,618 transcripts (**Figure 1A**) and 1,038 miRNAs (**Figure 1B**) were screeded in the ovary, and 2,968 differentially expressed mRNAs (DEGs) and 99 differentially expressed miRNAs (DEMs) were identified between the H and L groups. In the H vs. L comparison, there were 1,553 upregulated and 1,415 downregulated DEGs, and 80 upregulated and 19 downregulated DEMs (**Figures 1C, D**). All the identified DEGs and DEMs are presented in **Supplementary Table S2**.

**Table 1:** Transcriptome sequencing analysis for the identification of mRNAs and miRNAs in goat ovary.

Items	mRNAs	miRNAs
Clean reads	1,154,225,892	261,504,014
Total mapped reads	1,119,581,967	248,131,970
Total mapped rate (%)	96.99	94.88
Unique mapping reads	934,244,596	–
Unique mapping rate (%)	91.41	–

### Integrated Analysis of Differentially Expressed miRNAs and mRNAs

**Figure 2:**
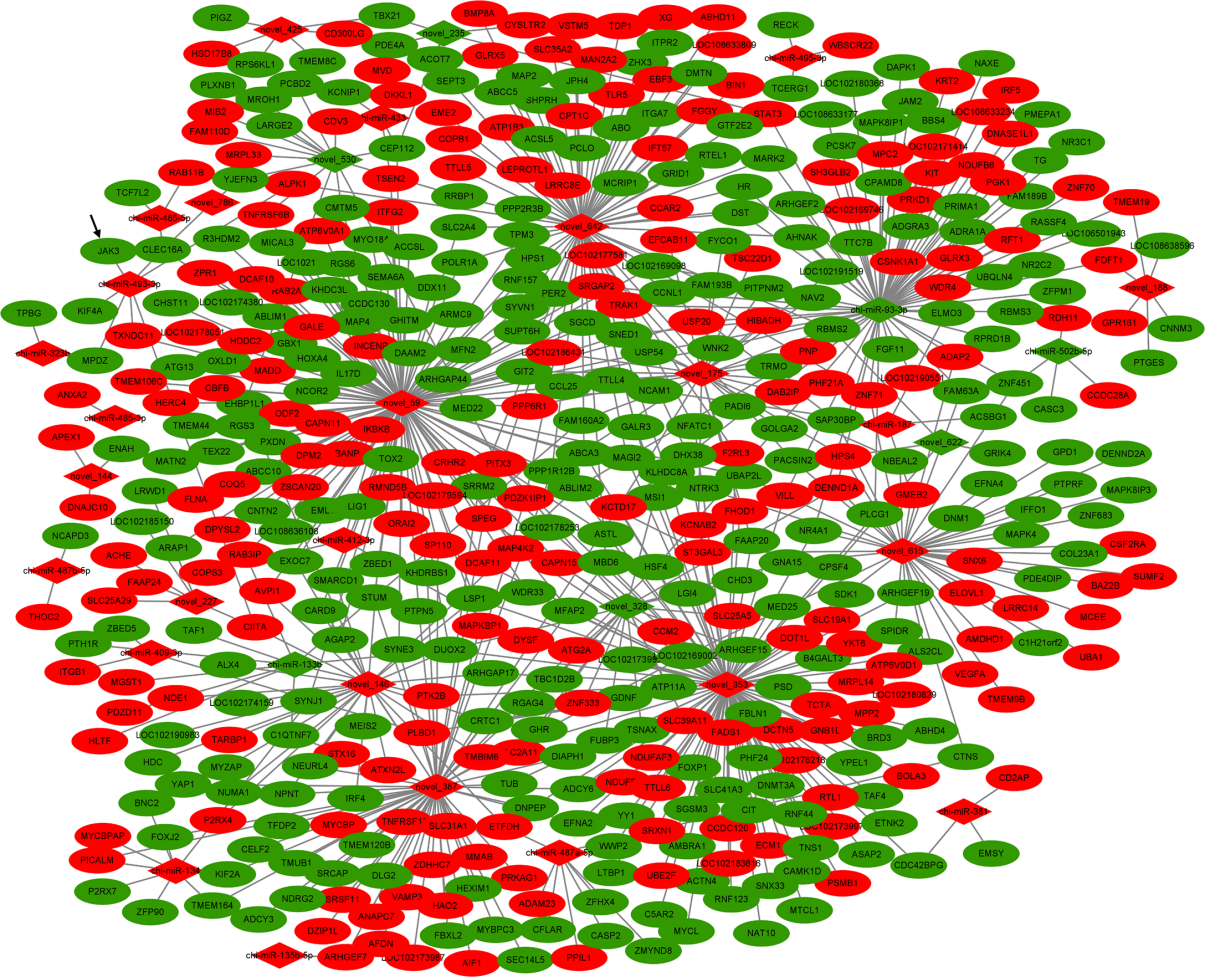
The DE miRNA–mRNA interaction network in the comparison. Red color: upregulated; Green color: downregulated.

The identified DEMs and DEGs from goat ovarian tissues of individuals were integrated to obtain the miRNA–mRNA network associated with goat prolific traits. A total of 671 miRNA–mRNA pairs were predicted in the H vs. L comparison (**Figure 2** and **Supplementary Table S3**), of which the core gene of network *DUOX2* is targeted by 5 miRNAs, namely, miR-133b, novel_146, novel_328, novel_353, and novel_59. The major function of miRNAs is to suppress the expression of target genes; therefore, the miRNA–mRNA pairs with negatively correlated expression levels are the focus of research. Among these DEG–DEM pairs, 386 pairs were negatively correlated in expression (*q*-value < 0.05), which might play key roles in the molecular mechanism of the goat prolific trait. In the network, miR-93-3p was a core miRNA with 73 miRNA–mRNA pairs, 29 of which showed a negative correlation. Another important core miRNA was miR-493-3p, which targets 7 genes including *JAK3, MPDZ*, and *CLEC16A*; these genes were closely related to the JAK-STAT signaling pathway, tight junction, and the PI3K-Akt signaling pathway. A previous study has shown that the PI3K-Akt signaling and JAK-STAT signaling pathways were associated with ovarian function, such as the primordial follicle recruitment, the proliferation of GCs, the survival of corpus luteum, and oocyte maturation (27, 28). *JAK3* was an important member of the membrane-related intracellular non-receptor tyrosine kinase protein family and participated in the JAK-STAT signaling pathway and PI3K-Akt signaling pathway. According to their potentially preserved functions, two regulators, miR-493-3p and *JAK3*, were chosen for further analyses in the studies related to goat prolific trait.

### The Analysis of Differentially Expressed Targeting Gene Functional Enrichment

**Figure 3:**
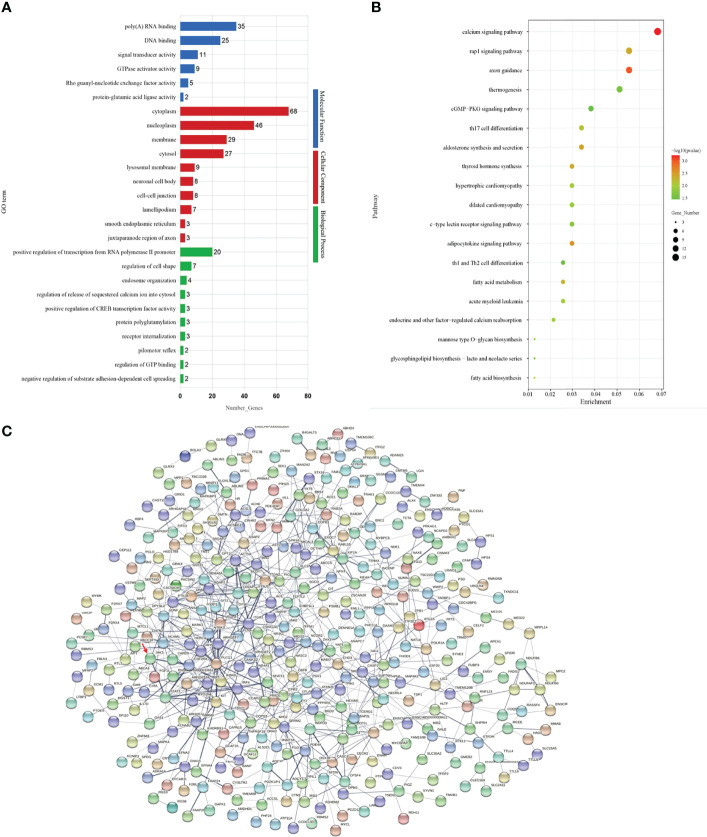
**(A)** The top 20 GO terms enriched in the comparison. **(B)** The top 20 KEGG pathways enriched in the comparison. **(C)** The protein–protein interaction (PPI) network of differentially expressed proteins from DEM–DEG pairs in the comparison.

In order to further investigate the functions of DEG–DEM pairs between the different fertility performance groups, the GO enrichment analysis was performed to reveal the biological process terms of the important DEGs (corrected *q*-values < 0.05). In this comparison, the associations were mainly with cytoplasm and nucleoplasm (**Figure 3A**). Then, the GO terms related to the reproduction trait are also displayed in **Supplementary Table S4**, including the positive regulation of the receptor signaling pathway by JAK-STAT, estrogen biosynthetic process, estrogen metabolic process, and ovarian follicle development. To further explore the function of DEMs–DEGs in regulation pathways for goat prolific trait, KEGG pathway analysis of miRNA–mRNA pairs was performed (**Figure 3B**). The enrichment of KEGG pathway in the pathway categories of the comparison is shown in **Supplementary Table S4**. In the comparison, the neurodegeneration–multiple diseases pathway (chx05022) and the calcium signaling pathway (chx04020) were the top two pathways. Among them, there eight signaling pathways were directly and indirectly associated with fertility trait, namely, the GnRH signaling pathway (chx04912), oocyte meiosis (chx04114), the PI3K-Akt signaling pathway chx04151), steroid biosynthesis (chx00100), ovarian steroidogenesis (chx04913), the JAK-STAT signaling pathway (chx04630), GnRH secretion (chx04929), and the estrogen signaling pathway (chx04915). A total of 21 DEGs were enriched in the eight pathways, including *JAK3, ADCY3, ADCY6, IKBKB, STAT*3, and other genes shown in **Supplementary Table S4**, which might be important candidates for further study.

Subsequently, the list of target genes extracted from the STRING database was used to construct the PPI network (**Figure 3C**). The PPI network on DEGs obtained from the H vs. L comparison contains 741 protein–protein nodes, of which 136 have a combined score of over 0.7. Thereinto, the key core nodes of the network included ITGB1, IRF4, ACTN4, CPSF4, NCOR2, KIT, FLNA, CSF2RA, COPB1, and JAK3, all of which were targets of DE miRNAs (shown in **Supplementary Table S5**).

**Figure 4:**
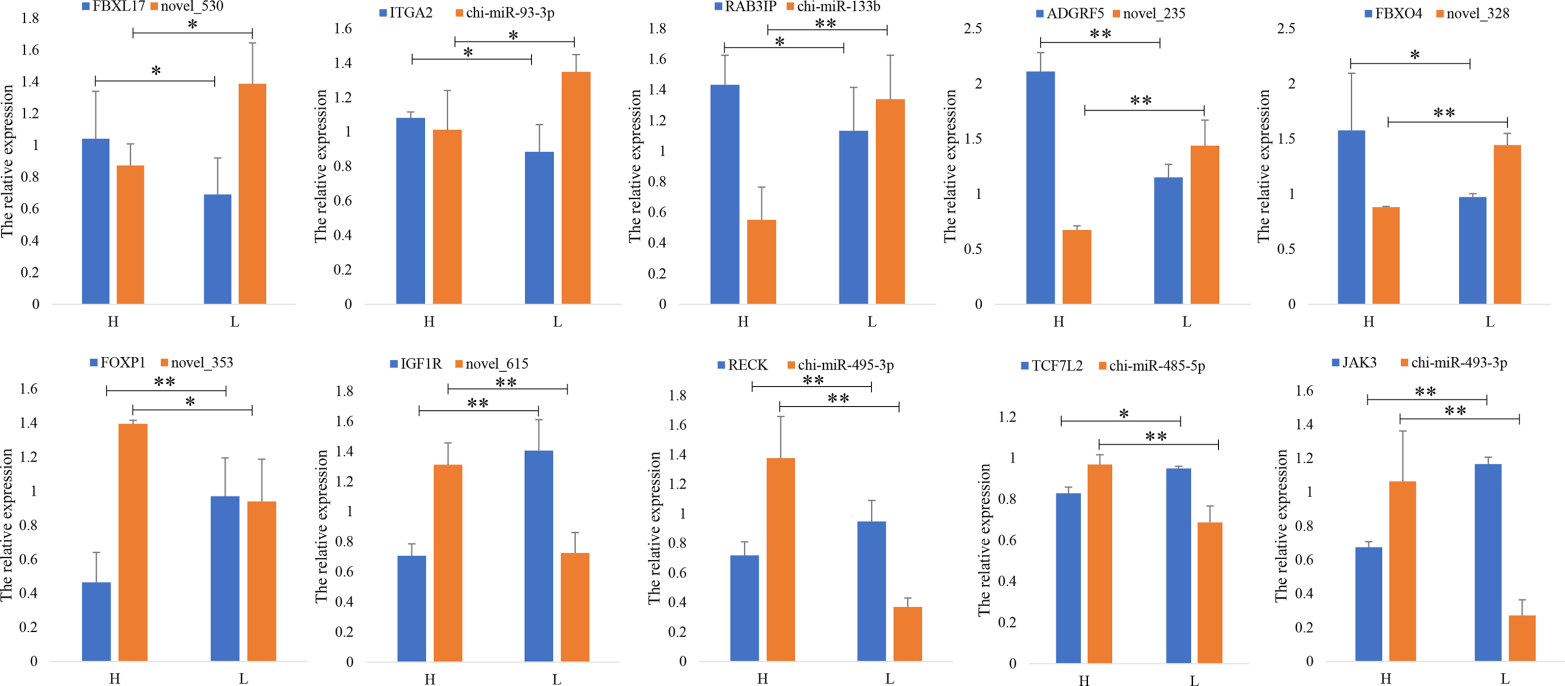
Verification of differential gene expressions. RT-qPCR quantifies 10 DEM–DEG pairs in high (H)- and low (L)-prolificacy goat ovary. RPL19 is used as an internal control. Values are expressed as mean ± SD of *n* = 3. *p < 0.05, **p < 0.01.

Ten DEG–DEM pairs from RNA-seq, namely, novel_530-*FBXL17*, chi-miR-93-3p-*ITGA2*, chi-miR-133b-*RAB3IP*, novel_235-*ADGRF5*, novel_328-*FBXO4*, novel_353-*FOXP1*, novel_615-*IGF1R*, chi-miR-495-3p-*RECK*, chi-miR-485-5p-*TCF7L2*, and chi-miR-493-3p-*JAK3*, were randomly selected for RT-qPCR validation. The results suggested that the expression levels of mRNAs and miRNAs agreed with the data from RNA-seq, indicating that the data from RNA-seq were plausible and that they have a negative correlation, indicating a potential interaction (**Figure 4**).

### Overexpression and Knockdown of JAK3 Regulated GC Proliferation

**Figure 5:**
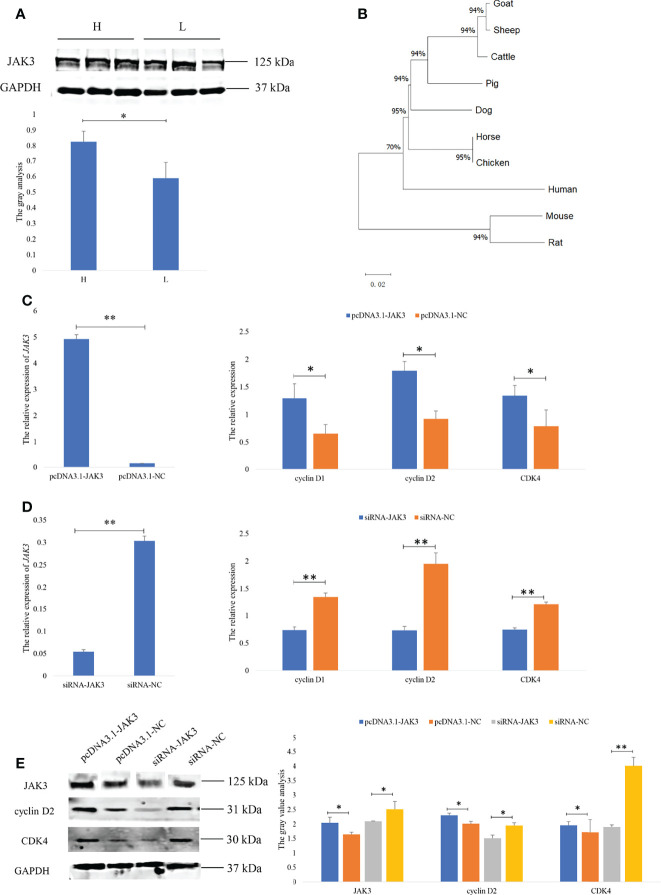
JAK3 promoted the granulosa cell proliferation. **(A)** The protein of JAK3 in goat ovary. H represents the high-prolificacy goats; L represents the lowprolificacy goats. **(B)** The phylogenetic tree analysis of JAK3. **(C, D)** The mRNA expressions of JAK3, cyclin D1, cyclin D2, and CDK4 in granulosa cells transfected with pcDNA3.1-JAK3/pcDNA3.1-NC or siRNA-JAK3/siRNA-NC for 48 h are quantified using RT-qPCR. RPL19 is used as an internal control. **(E)** The protein expressions of JAK3, cyclin D2, and CDK4 in granulosa cells transfected with pcDNA3.1-JAK3/pcDNA3.1-NC or siRNA-JAK3/siRNA-NC for 48 h are quantified using WB. GAPDH is used as an internal control. Values are expressed as mean ± SD of n = 3. *p < 0.05, **p < 0.01.

The proliferation of GCs is essential for ovulation in mammals. To reveal the molecular mechanism of the goat prolific trait, the function of *JAK3* in the goat GCs was furthermore studied. The Western blot results showed that the JAK3 protein expression was significantly higher in the H group than in the L group (*p* < 0.05) (**Figure 5A**), as opposed to *JAK3* mRNA expression. This result showed that the expression of JAK3 was regulated by the miRNAs in the post-transcript level. The phylogenetic tree analysis found that *JAK3* was highly conserved in different species and that the goat *JAK3* was first clustered with sheep, followed by cattle (**Figure 5B**). Hence, we supposed that the function of *JAK3* was similar to other species. To explore the role of *JAK3* in goat GC proliferation, *JAK3* overexpression and siRNA vectors were constructed. Overexpression of *JAK3* induced an increase in mRNA expression of the cell proliferation markers CDK4, cyclin D1, and cyclin D2 after 24 h post-transfection (**Figure 5C**). These results demonstrated that overexpression of *JAK3* promotes the proliferation of goat GCs. In contrast, we observed the opposite pattern in the knock-down experiment using si-JAK3 (**Figure 5D**). Western blot results showed that the protein expression of JAK3, cyclin D2, and CDK4 was consistent with mRNA expression (**Figure 5E**). Overall, these results demonstrated that *JAK3* promoted the proliferation of goat GCs.

### JAK3 Is a Target Gene of chi-miR-493-3p

**Figure 6:**
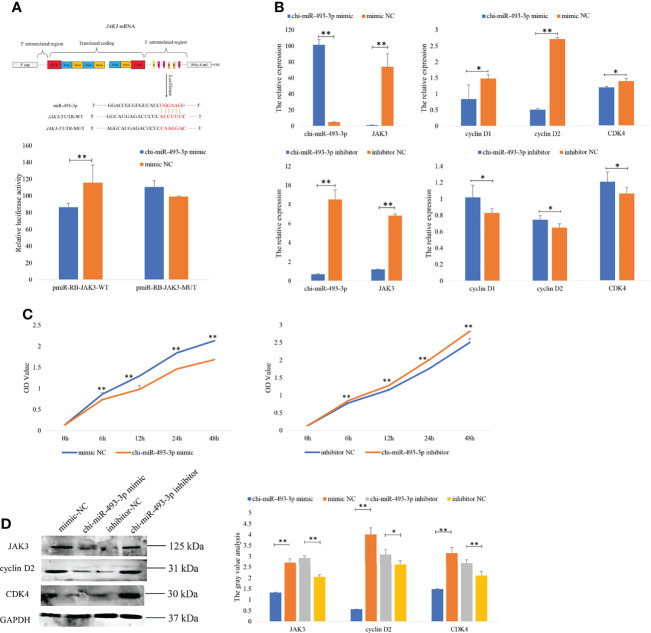
chi-miR-493-3p specifically targets JAK3 in goat granulosa cells. **(A)** Target sites for chi-miR-493-3p in the JAK3 3’-UTR and the construction of the luciferase expression vector (Luc) fused with the JAK3 3’-UTR. JAK3-3’UTR-WT represents the Luc reporter vector with the wild-type JAK3 3’-UTR; JAK3-3’UTR-MUT represents the Luc reporter vector with the mutation at the chi-miR-493-3p site in JAK3 3’-UTR. After HEK293T cells transfected chi-miR-493-3p mimic or mimics NC with WT-/MUT-Luc reporter vectors for 48 h, the relative luciferase activities are measured. The **(B)** mRNA expressions of *JAK3, cyclin D1, cyclin D2*, and *CDK4* in granulosa cells transfected with chi-miR-493-3p-mimic/-inhibitor or mimic/inhibitor NC for 48 h are quantified using RT-qPCR. *RPL19* is used as an internal control. **(C)** The CCK8 assay analysis in granulosa cells transfected with chi-miR-493-3p-mimic/-inhibitor or mimic/inhibitor NC for 48 h. **(D)** The protein expressions of JAK3, cyclin D2, and CDK4 in granulosa cells transfected with chi-miR-493-3p-mimic/-inhibitor or mimic/inhibitor NC for 48 h are quantified using WB. GAPDH is used as an internal control. Values are expressed as mean ± SD of *n* = 3. *p < 0.05, **p < 0.01.

Integrative analysis of DE miRNAs and mRNAs showed that *JAK3* was one of the targets of chi-miR-493-3p. To determine the function of the chi-miR-493-3p-JAK3 pathway, the dual-luciferase reporter assays were performed to validate whether there was a direct interaction between chi-miR-493-3p and *JAK3*. The luciferase activity of the *JAK3* wild-type plasmid with the chi-miR-493-3p mimic was significantly lower than that of the *JAK3* mutant plasmid (*p* < 0.01) (**Figure 6A**), supporting the hypothesis that *JAK3* is a direct target of chi-miR-493-3p. The expression of JAK3 mRNA and protein in goat GCs was determined after transfection with the chi-miR-493-3p mimic or chi-miR-493-3p inhibitor. The results showed significantly decreased mRNA (**Figure 6B**) and protein (**Figure 6D**) expression of JAK3 in the chi-miR-493-3p mimic transfected in GCs of goat (*p* < 0.01). In goat GCs transfected with the chi-miR-493-3p inhibitor, both mRNA and protein expression levels of JAK3 were significantly increased (*p* < 0.01) (**Figures 6B, D**). These results suggested that *JAK3* is a direct target of chi-miR-493-3p in goats.

### chi-miR-493-3p Suppressed GC Proliferation by Targeting JAK3

To investigate the function of chi-miR-493-3p in the proliferation of goat GCs, chi-miR-493-3p was overexpressed or inhibited in goat GCs. RT-qPCR results demonstrated that overexpression of chi-miR-493-3p in GCs significantly increased the expression level of chi-miR-493-3p, and reduced the expression of *JAK3*, as well as decreased the expression levels of proliferation-related genes (*CDK4, cyclin D1*, and *cyclin D2*) (**Figure 6B**). Moreover, CCK-8 assays indicated that the proliferative state of these GCs was depressed after transfection (**Figure 6C**). However, the GCs transfected with the chi-miR-493-3p inhibitor showed a significantly decreased level of chi-miR-493-3p mRNA and significantly increased levels of *JAK3*, *CDK4, cyclin D1*, and *cyclin D2* mRNA (**Figure 6B**). Subsequently, CCK-8 assays indicated that the proliferative state of these cells was promoted after transfection (**Figure 6C**). We also investigated the expression of JAK3, cyclin D2, and CDK4 proteins in GCs after transfected with the chi-miR-493-3p mimic and inhibitor and found that the protein levels of JAK3, cyclin D2, and CDK4 were all decreased in cells transfected with the chi-miR-493-3p mimic, whereas cells transfected with the chi-miR-493-3p inhibitor showed increased levels of JAK3, cyclin D2, and CDK3 proteins (**Figure 6D**). These results indicated that chi-miR-493-3p suppressed goat GC proliferation.

### The Expression of chi-miR-439-3p-JAK3 Promoted the E2 and PROG Secretion

**Figure 7:**
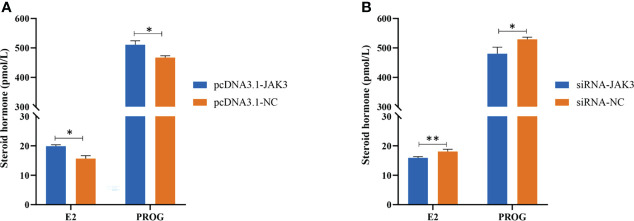
*JAK3* promotes steroid hormone synthesis. Goat granulosa cells are transfected with pcDNA3.1-JAK3 or pcDNA3.1-NC **(A)**, and siRNA-JAK3 or siRNA-NC **(B)**. After 24 h, cell-free supernatants are collected and estrogen (E_2_) and progesterone (PROG) secretions are measured in 50-μl supernatants using ELISA kits. Values are expressed as mean ± SD of *n* = 3. *p < 0.05, **p < 0.01.

After transfection of pcDNA3.1-JAK3 and siRNA-JAK3 in goat GCs for 24 h, the E_2_ and PROG levels in the cell-free supernatants were measured by ELISA to investigate the effect of JAK3 on synthesis of steroid hormones. JAK3 overexpressing cells showed enhanced secretion of E_2_ and PROG (**Figure 7A**), while inhibition of JAK3 reduced E_2_ and PROG production (**Figure 7B**). These results suggested that chi-miR-493-3p affected steroid hormone synthesis in goat GCs by regulating the expression of JAK3.

## Discussion

Reproduction is a complex series of physiological processes, and the hypothalamus–pituitary–ovary axis plays key roles in oocyte maturation and ovulation (29). In mammals, the ovary is both a gonad and an endocrine organ. In ovarian tissues, the primordial germ cells form the primary follicles and progress from the primary follicular stage to the mature follicular stage and finally to the production of functional oocytes (14). The oocytes are surrounded by somatic GCs and cumulus cells, whose interactions are crucial for oocyte formation. GCs can provide a number of factors to the metabolic pathways of the oocytes, such as specific amino acids, cholesterol, and steroid hormones (30–32). In most mammals (including mice and humans), ovarian sex steroids such as progesterone and estrogen are synthesized from the antral follicle and corpus luteum, which provide the foundation for oocyte maturation and ovulation (8). Goats are common herbivores that produce double kids, and the heritability of kidding rate is 0.11–0.14, with 2–6 ovulations in the estrus cycle (33). In recent years, to improve lambing numbers in goats, a variety of bioinformatics and molecular biology techniques have been applied to the study of high-prolificacy traits in goats. For example, Tao et al. conducted comparative genomic and transcriptomic analyses to determine genetic convergence in prolificacy between goats and sheep (34). However, the molecular mechanisms regulating multiple lambing in goats still need further study, especially combining functional genes with hormonal regulation.

It has been shown that miRNAs have potential molecular functions in regulating goat ovarian GCs, which are involved in the nourishment of oocyte and the secretion of steroid hormones (4, 35). In this study, we used RNA-seq to identify the potential miRNA–mRNA pairs, providing candidate functional pathways for studying high-prolificacy traits in goats. There were 671 miRNA–mRNA pairs predicted from the H and L group comparison, of which 386 pairs were found with negatively correlated expression. The miR-493-3p is an important core, which targets 7 genes, namely, *JAK3, MPDZ*, *ALPK1, CIITA, KIF4A, TXNDC11*, and *CLEC16A*. In a study of bovine oocytes, developmental competence revealed that the expression levels of components of the PI3K-Akt pathway in follicular cells carrying oocytes with different developmental capacities were found to relate to the molecular pathways participating in the access to oocyte capacity (27). During *Drosophila* oogenesis, the researchers found that AdamTS-A was a novel target for the JAK/STAT pathway by whole genome expression analysis and that it might play key functional roles in oogenesis (28). The PI3K-Akt pathway and JAK/STAT pathway are very important in the fertility trait of animals; however, the molecular mechanisms underlying the signal pathways still need to be determined. Janus kinase 3 (*JAK3*) is a member of the membrane-associated intracellular non-receptor tyrosine kinase protein family that regulates signaling initiated by cytokine and growth factor receptors *via* the JAK/STAT pathway (36, 37). In bovine GCs, *JAK3* is hormonally regulated and *LEPROTL1, INHBA*, and *CDKN1B* were identified to bind to *JAK3*, which may be involved in follicle growth by activation or inhibition of *JAK3* (38). *MPDZ* loss‐of‐function mutation caused severe hydrocephalus, resulting in acute morbidity and mortality in humans and mice (39). *ALPK1* (alpha-kinase 1) inhibits spontaneous breast mammary bi-lineage tumor-initiating cell differentiation and is a potential target for therapeutic development (40). GO term analysis revealed that these genes are closely associated with the JAK-STAT signaling pathway, PI3K-Akt signaling pathway, and tight junction. Otherwise, ten miRNA–mRNA pairs were chosen to validate the accuracy of the RNA-seq. The KEGG enrichment showed that eight signaling pathways were directly and indirectly associated with fertility trait, namely, the GnRH signaling pathway, oocyte meiosis, the PI3K-Akt signaling pathway, steroid biosynthesis, ovarian steroidogenesis, the JAK-STAT signaling pathway, GnRH secretion, and the estrogen signaling pathway. This study expands the amount of genetic information available, provides an overview of the physiological processes involved in these DMR–DEG pairs, and provides a first step towards understanding the function of mRNAs and miRNAs on goat ovaries with high and low prolificacy.

Among the various signaling pathways related to the PI3K/Akt signaling pathway, the regulation of folliculogenesis and oogenesis and their numerous branches appears to be the major non-gonadotropic factor regulating differentiation, growth, and survival of follicular cells (41, 42). Observations suggested that the various gene deletions in this pathway could lead to infertility and premature ovarian failure (POF), suggesting an essential role for this pathway in ovarian function. The LH surge triggered the oocyte maturation, ovulation processes, and luteinization when the gonadotropin causes a number of carefully designed signaling cascades that activate specific intracellular substrates. The LH surge stimulates PI3K/Akt, confirming the importance of the PI3K/Akt pathway in oocyte maturation (43). In this study, 11 enrichment DEGs were shown in the PI3K/Akt signaling pathway, namely, *JAK3, EFNA2, EFNA4, GHR, IKBKB, ITGA7, ITGB1, KIT, MAGI2, NR4A1*, and *VEGFA*. Analysis of the miRNA–mRNA network showed that *JAK3* was a target of chi-miR-493-3p, and we found that the mRNA and protein expression of JAK3 showed opposite trends in H vs. L comparison. Based on their potential functions, these two regulators, miR-493-3p and *JAK3*, were selected for further analyses in the studies concerning prolific trait of goats.

Subsequently, we specifically investigated the specific effects of *JAK3* on goat GCs *in vitro*. Using a gene expression profiling approach, *JAK3* was differentially expressed in GCs of bovine dominant follicles. The expression of *JAK3* was identified as one of the significantly downregulated in ovulatory follicles GCs after human chorionic gonadotropin (hCG) injection in bovine compared to dominant ovulatory follicles grown during the estrous cycle (44). The specific role of *JAK3* in GCs during follicular development such as from the small antral follicle before the LH surge to the preovulatory follicular stage was determined by activating or inhibiting the proliferation of target proteins in GCs (38). According to the obtained data and the survey results, one might hypothesize that the combination of *JAK3* and *LEPROTL1* could be involved in the process of follicle development through declining the negative feedback effects of potential *LEPROTL1* on GH signaling and improving the availability of *IGF-1* in the follicular growth. In this respect, GH has been proven to increase the secretion of IGF-1 from ovarian GCs *in vitro*, consistent with the results that GH signaling increase might regulate the function of the ovary *via* the secretion of IGF-1 from GCs (45). The results of our dual luciferase reporter assays showed that chi-miR-493-3p targets the *JAK3* 3’-UTR, which, in turn, inhibited its mRNA and protein expression levels, revealing that chi-miR-493-3p specifically targets *JAK3*. GC growth and follicular fluid formation were influenced by different steroid hormones, which, in turn, affect follicular development, including intrafollicular cell proliferation, apoptosis, and angiogenesis. E_2_ and PROG steroid hormones are well known to regulate the expression of genes associated with ovulation and luteal formation (46). Therefore, this study examined the effect of JAK3 on E_2_ and PROG and showed that JAK3 promoted the secretion of E_2_ and PROG. We also found that *JAK3* increased the mRNA levels of cyclin D1 and cyclin D2 and the protein levels of CDK4 and cyclin D2 in goat GCs. chi-miR-493-3p suppressed the mRNA levels of cyclin D1, cyclin D2, and CDK4 and inhibited the protein expressions of JAK3, cyclin D2, and CDK4 in goat GCs. Thus, we speculate that chi-miR-493-3p and JAK3 affect the proliferation of GCs and the secretion of E_2_ and PROG through cyclin D1, cyclin D2, and CDK4 cell proliferation-related genes.

In summary, 1,553 up- and 1,415 downregulated mRNAs, and 80 up- and 19 downregulated miRNAs were identified in high- and low-prolificacy goats using RNA-Seq. There were 671 miRNA–mRNA pairs predicted between the H and L comparison, of which 386 pairs were negatively correlated in expression. The analysis of GO terms and KEGG pathway showed that DEGs might take part in the regulation of ovarian development and growth. *In vivo*, chi-miR-493-3p directly targets *JAK3*, one of the downregulated DEGs, and regulated its expression in goat ovaries. chi-miR-493-3p caused GC proliferation by cell proliferation-related genes including *cyclin D1, cyclin D2*, and CDK4, and partially *via JAK3* reduction. *JAK3* also promoted the secretion of E_2_ and PROG in goat GCs. Our results provided a theoretical foundation and experimental evidence for the function of the chi-miR-493-3p-JAK3 pathway in ovarian development in goats.

## Data Availability Statement

The original contributions presented in the study are publicly available. These data can be found here: NCBI, BioProject, PRJNA731513, and PRJNA726268.

## Ethics Statement

All experimental procedures mentioned in this study were approved by the Science Research Department (responsible for animal welfare issues) of the Institute of Animal Sciences, Chinese Academy of Agricultural Sciences (IAS-CAAS) (Beijing, China). Ethical approval was provided by the animal ethics committee of IASCAAS (No. IAS2021-25).

## Author Contributions

Conceptualization: YL and MC. Methodology: YL, ZZ, SG, KL, PW, SC, SD, QH, and MC. Validation: YL, ZZ, SG, PW, YF, and KL. Formal Analysis: YL and MC. Investigation: YL, ZZ, SG, KL, and MC. Resources: YL, ZZ, SG, KL, XH, YJ, RL, and MC. Data Curation: YL, ZZ, SG, KL, and MC. Writing—Original Draft Preparation: YL and MC. Supervision: MC. Project Administration: YL, QH, and MC. Funding Acquisition: MC and QH. All authors contributed to the article and approved the submitted version.

## Funding

This work was financially supported by the National Natural Science Foundation of China (32102509), the Agricultural Science and Technology Innovation Program of China (CAAS-ZDRW202106 and ASTIP-IAS13), the China Agriculture Research System of MOF and MARA (CARS-38), the Major Science and Technology Project of Yunnan Province (202102AE090039), and the Basic Research Foundation Key Project of Yunnan Province (202001AS070002).

## Conflict of Interest

Authors SC and SD were employed by Wuhan Frasergen Bioinformatics Co., Ltd.

The remaining authors declare that the research was conducted in the absence of any commercial or financial relationships that could be construed as a potential conflict of interest.

## Publisher’s Note

All claims expressed in this article are solely those of the authors and do not necessarily represent those of their affiliated organizations, or those of the publisher, the editors and the reviewers. Any product that may be evaluated in this article, or claim that may be made by its manufacturer, is not guaranteed or endorsed by the publisher.

## Supplementary Material

Supplementary Table 2 The detailed information for DEGs and DEMs

Supplementary Table 3 The DEM–DEG pairs

Supplementary Table 4 The GO terms and KEGG pathway of DEM–DEG pairs

Supplementary Table 5 The results of PPI network analysis
