# Supplementary material for: Transcriptome Analysis Reveals Key miRNA–mRNA Pathways in Ovarian Tissues of Yunshang Black Goats With Different Kidding Numbers
Source: Front Endocrinol (Lausanne). 2022 May 19;13:883663. doi: 10.3389/fendo.2022.883663 (PMC9160789; doi:10.3389/fendo.2022.883663)
Supplement: Supplementary Table 2 — The detailed information for DEGs and DEMs [file DataSheet_1.zip › Supplementary Table S1 .DOCX]

**Table S1** The primer information of DEGs and DEMs

| Gene name | Primer sequence (5’-3’) | Product length (bp) | Tm (℃) |
| --- | --- | --- | --- |
| *JAK3* | F: TTCCCACAGCAGTCTTCGAG | 281 | 60 |
|  | R: AGCCCCGATAAATCTTGGTG |  |  |
| *TCF7L2* | F: CACCGCCCGAACTTATCTTC | 126 | 58.5 |
|  | R: AGGGGGCACTGAACGATGT |  |  |
| *RECK* | F: CTGGTGCTTGTTGCCCATTAT | 140 | 60 |
|  | R: AGACACGTGCATGCGGATC |  |  |
| *FOXP1* | F: AAACCACGGGCAACAATCAC | 279 | 60 |
|  | R: TATCGTCCAGCGCATGCTC |  |  |
| *IGF1R* | F: CTCTCTCTCGGGGAATGGGT | 122 | 59.5 |
|  | R: CCACTATCAACAGAACCGCAAT |  |  |
| *ITGA2* | F: TGGCATAGCAGTTCTTGGGTAC | 121 | 58.7 |
|  | R: ACTGCACCCAACGTCAGAATAT |  |  |
| *RAB3IP* | F: GCAAAGAGGCTGACTTATCCC | 257 | 58 |
|  | R: ACATTTTTTTGGTCCTCCGC |  |  |
| *ADGRF5* | F: AGATTTTCCCGAGTTTTACGG | 109 | 59 |
|  | R: AGCTCTGCCTGTACGATTGG |  |  |
| *FBXO4* | F: CTGGTTTGCCTCAGAGGCAG | 205 | 61 |
|  | R: ACACTGTACCGGCTCCCTTG |  |  |
| *FBXL17* | F: ACCAGTCGGTGAAGGCGTT | 261 | 60 |
|  | R: GTCCTTCCTTGGCGATAACCT |  |  |
| *PRL19* | F: ATCGCCAATGCCAACTC | 154 | 60 |
|  | R: CCTTTCGCTTACCTATACC |  |  |
| *Cyclin D1* | F: GCCACAGACGTGAAGTTCATTT | 156 | 60 |
|  | R: CGGGTCACATCTGATCACCTT |  |  |
| *Cyclin D2* | F: ATGTGGATTGCCTCAAAGCC | 152 | 58 |
|  | R: CAGGTCGATATCCCGAACATC |  |  |
| *CDK4* | F: GAGCATCCCAATGTTGTCAGG | 172 | 59 |
|  | R: ACTGGCGCATCAGATCCTTT |  |  |
| miR-485-5p | F: GGCTGGCCGTGATGA |  | 60 |
| miR-495-3p | F: CGCAGAAACAAACATGGTG |  | 60 |
| miR-493-3p | F: GCAGTGAAGGTCTACTGTGT |  | 60 |
| novel_353 | F: GCCCAGAGCCGTAGAC |  | 60 |
| novel_615 | F: GCAGTGAGGACAAGGGAC |  | 60 |
| miR-93-3p | F: CTGCTGAGCCAGCAC |  | 60 |
| miR-133b | F: GTTTGGTCCCCTTCAACC |  | 60 |
| novel_235 | F: GTTGGTCTTCATTGTTGAGCA |  | 60 |
| novel_328 | F: CAGCTGAAGGCTGTCC |  | 60 |
| novel_530 | F: CGCGAGGCCGGT |  | 60 |
| U6 | F: CAAGGATGACACGCAAATTCG |  | 60 |
